# The systematic evaluation of an embodied control interface for virtual reality

**DOI:** 10.1371/journal.pone.0259977

**Published:** 2021-12-07

**Authors:** Kenan Bektaş, Tyler Thrash, Mark A. van Raai, Patrik Künzler, Richard Hahnloser

**Affiliations:** 1 Institute of Computer Science, University of St. Gallen, St. Gallen, Switzerland; 2 Department of Biology, Miami University, Oxford, OH, United States of America; 3 LimbicLife AG, Zürich, Switzerland; 4 University of Zürich and ETH Zürich, Zürich, Switzerland; National Tsing Hua University, TAIWAN

## Abstract

Embodied interfaces are promising for virtual reality (VR) because they can improve immersion and reduce simulator sickness compared to more traditional handheld interfaces (e.g., gamepads). We present a novel embodied interface called the Limbic Chair. The chair is composed of two separate shells that allow the user’s legs to move independently while sitting. We demonstrate the suitability of the Limbic Chair in two VR scenarios: city navigation and flight simulation. We compare the Limbic Chair to a gamepad using performance measures (i.e., time and accuracy), head movements, body sway, and standard questionnaires for measuring presence, usability, workload, and simulator sickness. In the city navigation scenario, the gamepad was associated with better presence, usability, and workload scores. In the flight simulation scenario, the chair was associated with less body sway (i.e., less simulator sickness) and fewer head movements but also slower performance and higher workload. In all other comparisons, the Limbic Chair and gamepad were similar, showing the promise of the Chair for replacing some control functions traditionally executed using handheld devices.

## Introduction

*inhabito, ergo sum*

Virtual reality (VR) requires control interfaces to translate the movements of the user into movement through a virtual environment that is presented on a visual display. However, most current VR technologies do not provide a convincing sensation of self-motion in the absence of actual motion (i.e., vection) [1, 2]. This shortcoming is primarily attributable to the discrepancy between the level of immersion that can be induced by a particular VR system and users’ experience of presence in the virtual environment [3]. As a result, users cannot inhabit a virtual environment as they can inhabit a real environment [4].

According to Dourish, *embodied interaction* necessitates control interfaces that encourage a high level of engagement with the virtual environment during which the system reacts to users’ actions in a meaningful way [4]. Several interfaces have been proposed that encourage (full or partial) body movements while users stand [5], lie [6], or sit [7]. For example, while sitting on a chair, users’ body movements can be used to activate a control interface (i.e., motion cueing) for the simulation of locomotion in a virtual environment [9]. VR systems that allow a user to physically walk [10, 11] or otherwise take advantage of body-based information can enhance a user’s navigation performance [12, 13] and spatial updating [14, 15]. Navigation in a virtual environment can be seen as a combination of two main tasks: wayfinding (i.e., self localization with respect to a destination) and locomotion (i.e., to maneuver with respect to a target or obstacle) [16]. Interfaces that rely on body-based information have been found to be especially beneficial for navigating through large-scale virtual environments, including complex buildings and cities [17]. For example, Kruijff and colleagues compared a joystick control interface to an interface that mapped users’ leaning movements to translations through the virtual environment [18]. Such leaning-based interfaces have been found to improve vection [18] and presence [5]. Our work extends these findings by addressing whether leg movements performed in a sitting position can result in better navigation performance and yield better user experience than a conventional gamepad.

**Fig 1:**
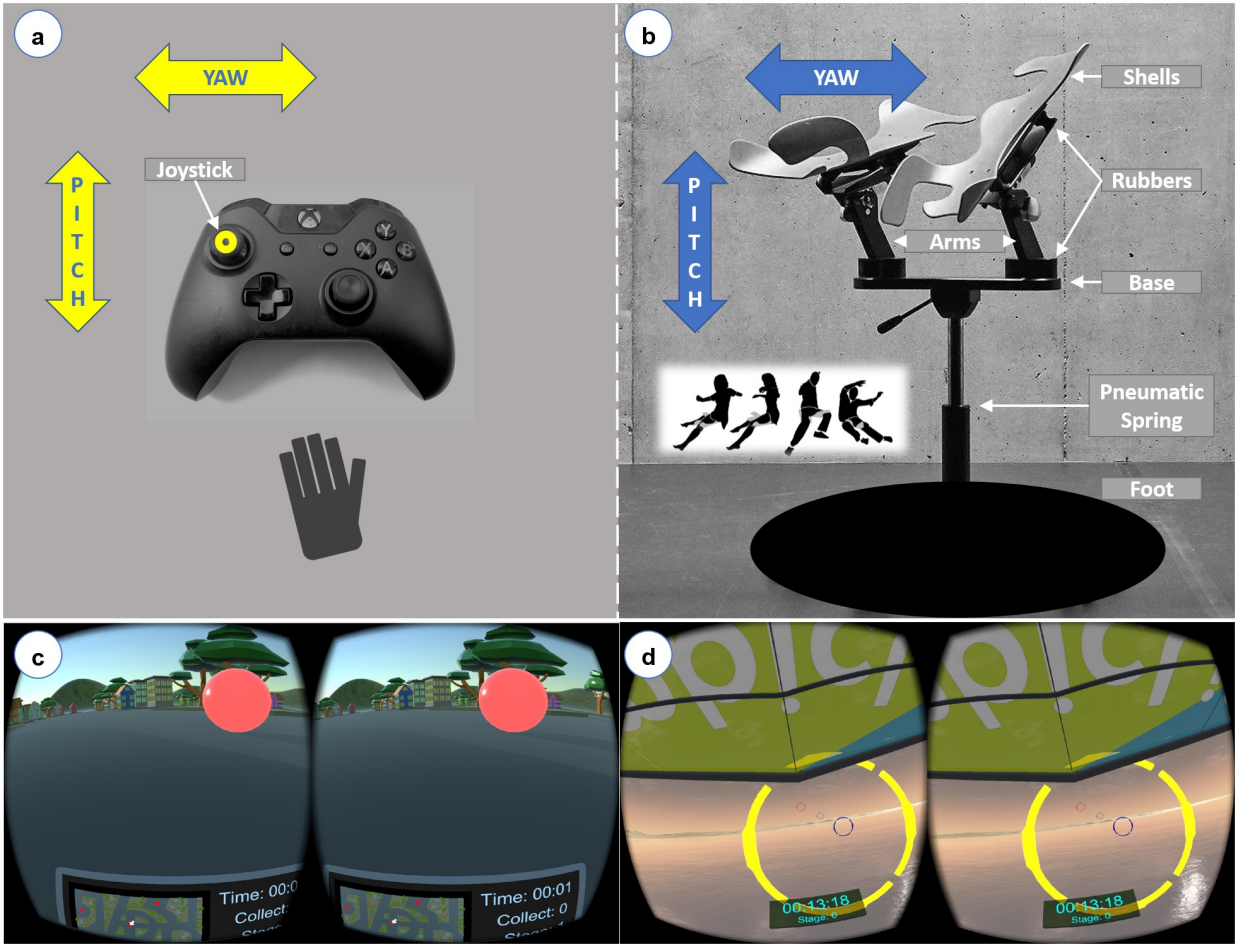
Two control interfaces (a,b) and two VR experiments (c,d). (a) The gamepad is a conventional device with which pitch and yaw movements can be controlled by hand. (b) On the Limbic Chair, the pitch and yaw of users’ legs are translated into movements through the virtual environment. Images of (c) the city navigation scenario and (d) the flight simulation scenario as displayed in the Oculus Rift [8].

We propose a new embodied interface, the Limbic Chair, that provides two degrees of freedom to each leg to simulate locomotion in VR. In two experiments, we aimed to answer the following research question: How suitable is the Limbic Chair for tasks in virtual environments that are typically performed with conventional control interfaces? We employ a different type of task in each experiment (i.e., city navigation and flight simulation) and measure several variables related to locomotion performance and user experience (see Fig 1). In both experiments, the participants were asked to pilot a virtual vehicle, but they were not expected to walk, jump, or pick up objects from the ground. These experiments were conducted under the permission of the ETH Zürich Ethics Commission (Proposal Number: 2017-N-57).

This paper has two original contributions. First, we introduce a novel embodied interface for human-computer interaction. The Limbic Chair is an ergonomic apparatus with great potential for applications that make use of human movements as spatial inputs. Second, we perform a thorough user evaluation of the suitability of this chair for embodied interactions with virtual environments. The evaluation is composed of two user experiments that systematically compare this new interaction device with a more conventional gamepad.

## Related work

### Control interfaces for navigation

All virtual reality technologies require a control interface to mediate between the user’s actual, physical movements and the movements of the user’s avatar/camera through the virtual environment. Control interfaces can vary with respect to the types of physical movements performed by the user, the types of movements that are possible in the virtual environment, and the mapping between physical and virtual movements. Physical movements can range from fine-grained hand movements to full body movements, and virtual movements typically involve walking, riding in a vehicle, or flying through the air. Innovative control interfaces are also being explored for performing novel types of actions and tasks such as remotely controlling a robot [19, 20]. However, the mapping between physical and virtual movements may become difficult for the user to learn if the physical and virtual movements require very different effectors (i.e., the body parts used to produce an action). For example, handheld control interfaces such as gamepads are manipulated using physical movements of the thumbs but may be used to simulate the flight of a bird. While these mappings may be more difficult to learn initially, the modern world is filled with such mappings. For example, the most typical mapping for producing the movement of a pointer on a computer screen is a handheld mouse with one or two buttons. In experimental contexts, it has been shown that handheld interfaces, such as a conventional joystick or a gamepad, can produce realistic walking behavior [21] and can lead to better navigation performance [5], spatial updating performance [9], and comfort and precision [22] than more embodied interfaces. In general, there appears to be a trade-off between a user’s familiarity with a control interface and the extent to which the mapping between physical and virtual movements is intuitive, allowing for embodied interaction [23].

### Chairs as embodied control interfaces

Immersive virtual environments allow for embodied interaction insofar as users can find a sense of self-location, agency, and body ownership in those environments [24, 25]. For an embodied experience, a meaningful spatial relationship between the user’s self, the user’s physical body, and the environment must be maintained. The *Self-location* can be maintained with an egocentric visuospatial perspective that is supported by other vestibular and tactile feedback. During active control movements in a virtual environment, the simulated and actual consequences of a user’s movements must match so that the user can feel a *Sense of Agency* with those actions. Any discrepancy between the actual movements and feedback from the actions (e.g., visual) might interfere with the subjective perception of agency. Finally, *Body Ownership* affects embodiment and is related to the level at which a user feels in possession of a virtual body through multi-sensory feedback (e.g., visual appearance of the avatar, tactile).

For navigation tasks in virtual environments, existing control interfaces include a rich set of body-aware input techniques that vary from hand-based interaction to whole-body interaction [26]. According to Kilteni and colleagues, a sense of embodiment can be enhanced if the user’s actual motion is mapped to its virtual representation through the real-time tracking of limbs or full-body movements [24]. Chairs are common embodied control interfaces for VR applications because they allow leaning-based motion cuing and are multi-functional and intuitive to use [9, 22, 27–32]. The chair-like interfaces may increase immersion and mitigate the symptoms of simulator sickness by relying on user’s leaning and active motion cuing [22, 33] to simulate proprioceptive and vestibular cues [1, 9]. Typically, these embodied control interfaces are equipped with passive sensors such as accelerometers and inertial measurement units (IMUs) that translate the actual movements of the user’s lower extremities (i.e., from feet and legs to pelvis) to simulated locomotion in the virtual environment. Thus, chair-like interfaces have the potential to stimulate body ownership and the senses of self-location and agency.

For example, NaviChair [9, 22] is a stool (without a backrest) that allows users to control forward and backward locomotion in a virtual environment as the user leans in the desired direction. The rotational movements of the NaviChair to the left or right are mapped to simulated rotations along the yaw axis. In [22], the authors compare the NaviChair with other leaning-based interfaces such as the *Head-directed Interface* that employs a head-mounted display for simulated translation and rotation movements and the *Swivel Chair* that combines a conventional chair with an external tracking system. Similarly, Probst and colleagues employ a commercially available flexible chair on which user’s leaning, rotational, and bouncing movements are tracked by the inertial measurement sensors of a smartphone that is attached to the backrest of the chair [29]. These control interfaces were tested by enabling users to navigate on the ground along two dimensions of the virtual environment [9, 22], or to maintain an embodied interaction with various desktop applications [29]. However, such interfaces can be used in 3D scenarios such as flight simulation.

### Embodied control interfaces for flight scenarios

Historically, flight simulation has been relevant for many VR applications, including the training of real pilots to the entertainment of users without extensive training [34, 35]. In these applications, non-embodied and conventional joystick controllers are often employed as the primary control interface. Indeed, in the literature, there are only limited number of embodied control interfaces that have been developed for or employed in flight simulation scenarios.

For example, Birdly [6] allows its user to embody a bird of prey by means of multisensory stimulation, including proprioceptive (i.e., the arm and leg movements correlate with the wings of the bird), tactile (e.g., headwind simulated by a fan), audio, and olfactory feedback. Birdly displays the virtual environment on a head-mounted display from the bird’s visual perspective. Cherpillod and colleagues present one of the few experimental studies with the Birdly [36]. In a fixed-wing drone piloting scenario, these researchers found that Birdly provide a more natural experience to untrained users compared to a conventional remote controller.

In a flight simulation scenario, Hashemian and colleagues compared user performance and experience with a gamepad controller and three leaning-based interfaces including two hybrid controllers (i.e., a combination of a gamepad controller with a chair-based interface) and the hands-free HeadJoystick (i.e., a combination of a head-mounted display and a swivel chair) [33]. Their findings show the benefits of leaning-based interfaces for flight scenarios in terms of accuracy, precision, ease of learning, ease of use, usability, long-term use, presence, immersion, sensation of self-motion, workload, and enjoyment.

### Simulator sickness

Studying new control interfaces for VR is important because any gap between immersion and presence may result in a reduced sense of embodiment [24] and even cause adverse effects such as simulator sickness [37]. Simulator sickness is a common side effect of VR that can occur when there is a discrepancy between visual feedback from the display and vestibular signals. According to Riccio and Stoffregen, simulator sickness is strongly connected with postural instability [38], and notably, this postural instability (or body sway) can be used to predict simulator sickness before it actually occurs [39]. The measurement of body sway can be complemented by questionnaires such as the Simulator Sickness Questionnaire (SSQ) originally used by Kennedy and colleagues [40]. The SSQ includes a list of questions related to specific symptoms of simulator sickness (e.g., nausea, fatigue, and headache).

### Research gaps

The precise advantages and disadvantages of conventional interfaces (e.g., gamepads) and embodied interfaces (e.g, chairs) can vary among studies [22, 33]. Thus, to study the potential benefits of a new embodied interface in a series of user experiments, the gamepad can be considered as a *gold standard* (i.e., control case). Previous research has compared leaning-based interface to gamepads primarily in terms of ground navigation using questionnaires tailored to their particular studies. In two experiments, the present paper aims to compare an ergonomic chair interface with separable leg movements to the gamepad in terms of both ground navigation and flight scenarios using standardized questionnaires.

## Experiment 1: City navigation

Embodied control interfaces can improve navigation performance [14, 15], vection [18], and presence [5]. There is also evidence that conventional interfaces (e.g., gamepads, joysticks) improve navigation [5], spatial updating performance [9], and comfort and precision [22]. Inspired by [22], our primary goal for Experiment 1 is to compare a new embodied control interface with a traditional control interface by exploring its suitability for VR in terms of user experience during navigation through a virtual city. Methodologically, we slightly diverge from [22] by employing standardized questionnaires for measuring user experience and body sway for measuring simulator sickness. The advantage of standardized questionnaires over questions tailored to a particular study is that the results can be compared across studies [25]. We also measure body sway as another indicator of simulator sickness. In line with previous research [22, 32, 41], we augmented our evaluation with a content analysis of oral feedback provided by the participants at the end of the experiment.

### Participants

Eighteen people participated in this experiment. The data from four participants were incomplete because they interrupted the experiment. The data from 14 participants (5 female and 9 male, aged 22 to 43) were included in the analysis. The participants had normal or corrected-to-normal vision and were informed of the potential risk of simulator sickness. The participants had no prior experience with the Limbic Chair, and only two participants had no prior experience with a gamepad. All participants received 25 CHF as compensation.

### Apparatus and materials

A workstation (Intel Core i5 4690K @ 3.50GHz CPU, EVGA GTX 970 Superclocked ACX 2.0 (4GB) GPU, and 16 GB RAM) was used to render the virtual environment in the Unity [42] game engine. The virtual environment was displayed through an Oculus Rift VR headset at a resolution of 1080 × 1200 pixels across a 110° (total) field of view with a refresh rate of 90 Hz.

The two control interfaces were a handheld Microsoft Xbox-One gamepad and the Limbic Chair (Fig 1). The gamepad’s dimensions are 15.50 × 6.10 × 10.80 cm. The gamepad weighs approximately 230 grams and costs about 60 CHF. During the experiment, only the left analog joystick of the gamepad was used to enable movement along the pitch and yaw axes. In the resting state of the gamepad, there was a *dead zone* surrounding the central position of the joystick where it does not produce any visual movement on the display.

The Limbic Chair [43] is composed of two ergonomic shells, two mechanical arms, a base, a pneumatic spring, and a static foot (Fig 1b). Each shell rests on one of the mechanical arms that is vertically fixated to one end of the base. We installed rubber elements at the joints that attach the shells to the arms and the arms to the base. The height of the chair can be adjusted using the pneumatic spring. The design of the Limbic Chair does not include a backrest or any armrests. While using the Limbic Chair, users’ hands may remain free. To achieve a sensation of smooth and continuous control, we did not build a dead zone into the Limbic Chair’s signal in the firmware itself or in the experimental software. Similar to the other embodied control interfaces listed in [22], the space requirement of the Limbic Chair does not exceed that of a conventional static or swirl chair used in home or office environments. The diameter of the Limbic Chair’s foot is 50 cm, and the height of the chair can be adjusted between 60 and 80 cm. The Limbic Chair weighs 8.9 kilograms and costs 2950 CHF.

The Limbic Chair measures the pitch and yaw of each leg and translates these measurements into movements in forward, left, and right directions. Because the legs are resting on two separate shells, the Limbic Chair provides users’ legs more freedom to move while sitting compared to other motion cueing interfaces that also take advantage of users’ lower body movements (see [22] for a review). Having separate shells also allows users to engage individual leg movements in different actions. For example, during a navigation task, the movement of a single leg outwards along the yaw axis can be assigned to maneuvering in the respective direction. A similar mapping would also be suitably used in a browsing task for swiping or selecting images in coordination with the individual leg movements.

On the Limbic Chair, yaw is measured by an optical encoder mounted to the pivot point underneath the arm of each shell. Because of the rubber elements in the chair, pitch and roll cannot be measured by purely mechanical means and are measured using inertial measurement units (IMUs; a combination of accelerometers and gyroscopes). The yaw angles are each measured by a CUI AMT102 optical encoder [44] with 2048 grooves per full revolution. The yaw angles have an angular resolution of 360°/2048 ≈ 0.2°. During pilot testing, we have found this resolution to be sufficient for users to perceive a smooth continuous on-screen motion while moving the shells with their legs. The IMUs were a pair of Invensense MPU6050s mounted underneath each of the shells. In short, gyroscopes measure angular momentum precisely with low noise but with significant drift. On the other hand, accelerometers measure the angle without drift but with a significant amount of noise. The gyroscope signal is integrated over time, and the accelerometer signal is used to compensate for drift. Because the accelerometer relies on gravity for measuring the angle of movement, it cannot compensate for yaw drift along the horizontal plane. We have developed a relatively simple fusion algorithm of the gyroscope and accelerometer signal, but the details of this algorithm are beyond the scope of the present paper. The combined signals were transmitted to the workstation at a constant rate of 200 Hz. With the Limbic Chair as an input device, the fusion algorithm results in a VR experience that is free of detectable lag, jitter, or drift.

The output signals from both the gamepad and the Limbic Chair for each analog axis consist of a 16-bit integer signal (i.e., values between -32767 and 32767). This precision is specific to the (Xbox-One) controller used in this experiment. Most other controllers have a lower resolution for the analog axes. The joystick’s output signal was normalized to a floating point value between -1 and 1 at the extreme positions. Releasing the joystick physically resets its position to zero. The Limbic Chair’s signal is normalized to a floating point value between -180° and 180° for each axis and directly corresponds to the physical angle of the shells themselves. The zero value for each axis corresponded to the initial angle of the shells when the experiment started. For example, an offset from an initial angle of 180° resulted in the chair sending a value of 32767 for that axis to the workstation, which then converts it back to a floating point value of 180°.

**Fig 2:**
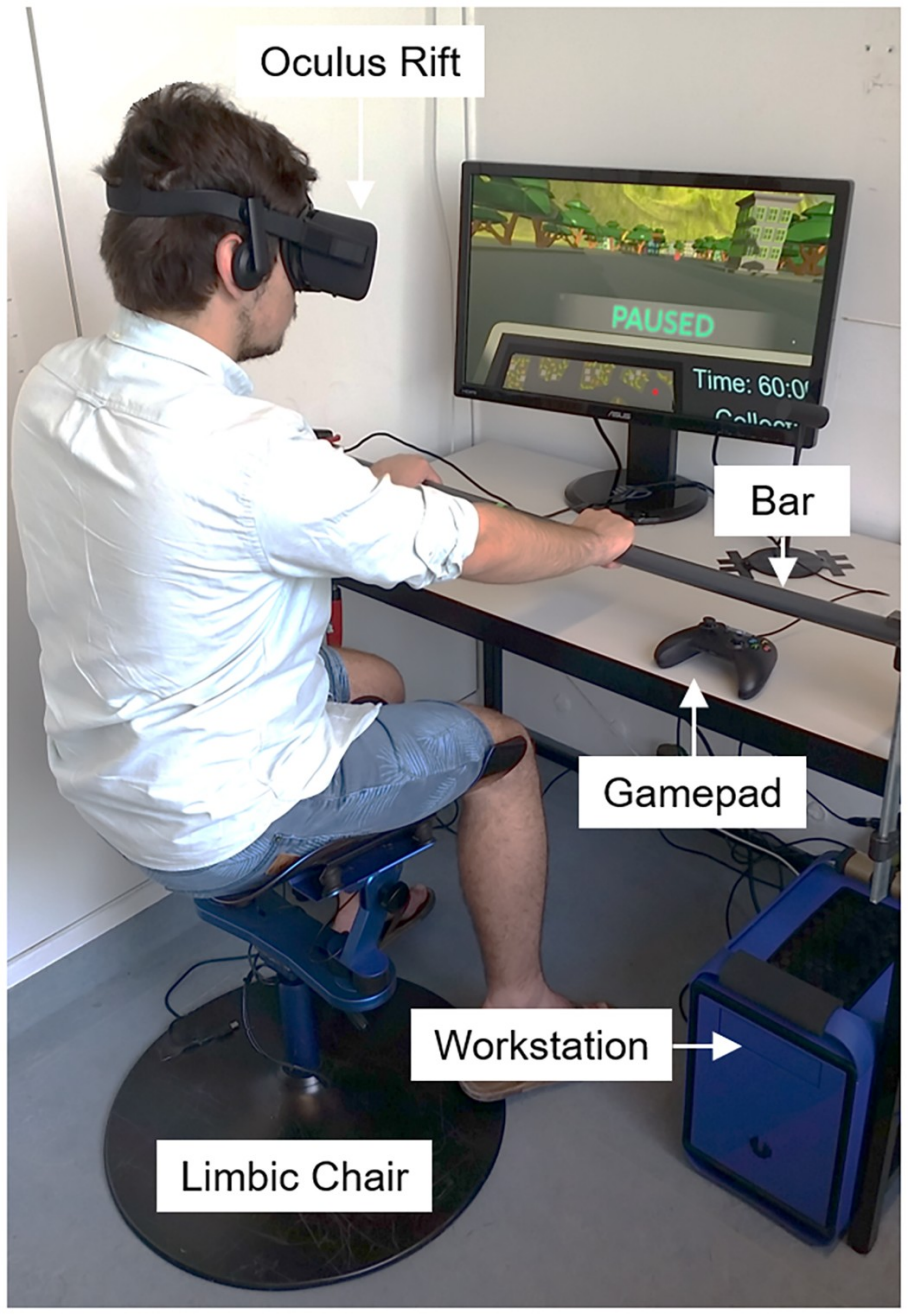
Our VR system is composed of an Oculus Rift, a Limbic Chair, a gamepad, a workstation, and a horizontal bar fixed to the workbench. The user can move his left and right legs independently while balancing on the chair.

Units of distance are arbitrary in a virtual world, but a spatial unit of 1 in the Unity game engine, referred to as *units* here, roughly corresponded to 1 meter in the real world. For example, moving the joystick forward resulted in a forward movement in the virtual environment at a constant speed of 50 units per second. We kept movement speed constant to prevent speed from becoming a confounding variable for the assessment of participants’ performance and simulator sickness. Before starting the experiment, we measured the height of participants’ heads from the floor while they were sitting on the chair (see Fig 2). This measurement was then used to adjust their head position in the virtual environment.

In the following description, we define *yaw* as the horizontal angle and *pitch* as the vertical angle relative to the virtual floor. *dT* is the time elapsed between the frames in which all inputs, virtual environment parameters, and graphics are updated by the Unity. The equations below represent code-snippets of our implementation of the input mapping for the virtual environment translated to equations. In this experiment, the participant either remains stationary or moves forward at a constant speed of 50 units per second. To move forward, the participant pushes the joystick forward (pitch) to any degree greater than the deadzone. For the Limbic Chair, in order to move forward, the participant moves their knees apart, resulting in a horizontal angle between the shells that is greater than zero. Rather than using forward pitch to move the participant forward, this modality was chosen because it was perceived as more intuitive during pilot testing. A systematic comparison between different modes and sensitivities for controller mapping would be interesting for future research.

The yaw movements of the joystick were mapped to left and right horizontal turns in the virtual environment via Eq 1:

$$\begin{array}{c} yaw_{i+1}=yaw_{i}+100\times Joy_{x}\times dT, \end{array}$$ (1)

where *i* represents the index of the update frame and *Joy*_*x*_ is the left/right axis of the joystick. Values for *Joy*_*x*_ lie between -1 and 1.

Analogous to the joystick, the yaw movements of the Limbic Chair’s shells were mapped to horizontal turns in the virtual environment via Eq 2:

$$\begin{array}{c} yaw_{i+1}=yaw_{i}+5\times \left(LeftLeg_{x}+Rightleg_{x}\right)\times dT, \end{array}$$ (2)

where *LeftLeg*_*x*_ and *Rightleg*_*x*_ are the left and right shells’ rotation angles (i.e., yaw angles) in degrees, respectively. For both equations, the horizontal turn continues as long as the yaw movement is maintained. The constant multipliers in Eqs 1 and 2 (respectively 100 and 5) were determined empirically based on pilot testing. The maximum turning rate with the gamepad was 100°/*s* and could be achieved by holding the joystick all the way to the left or right. The same turning rate could be achieved with the Limbic Chair by turning both legs 10° away from their starting positions in the same direction along the horizontal plane. In order to increase immersion and proprioception, we installed a horizontal bar fixed to the workbench (Fig 2). This also allows participants to orient their torso with respect to their movement through the virtual environment.

We recorded the participants’ head movements during each session using the head-angle output from the Oculus Rift headset. These measurements are the same as those that are used by various games to determine a player’s in-game viewpoint. We recorded all three head angles (pitch, yaw, and roll) during each update frame as described above. We then integrated the total angle displacement over the entire (active) game-time per controller as a measure of total head-movement.

Before and after using each controller, we used a Nintendo Wii Balance Board (WBB) to measure participants’ body sway (cf. [45]). Sensors on the WBB detect changes in the body’s center of pressure at a 30-50 Hz sampling frequency in the side-to-side and front-to-back directions [46]. We collected the body sway data with BrainBloX software [47] installed on a laptop computer and connected to the WBB via bluetooth.

**Fig 3:**
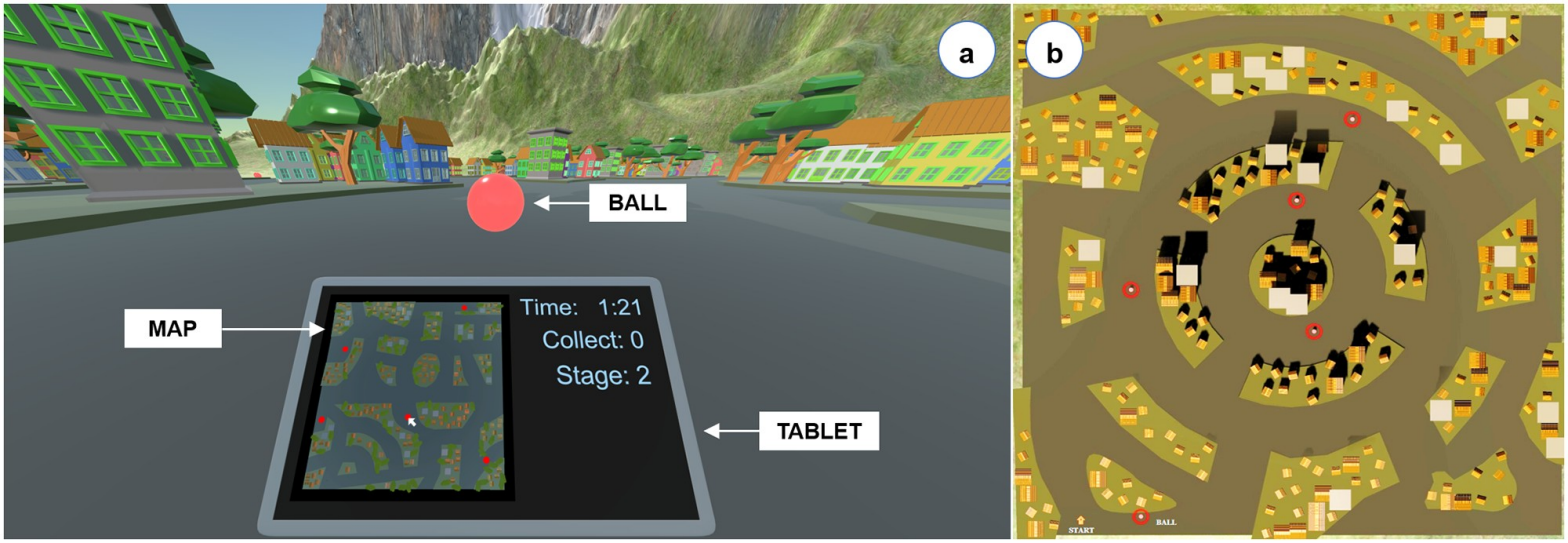
(a) A screenshot of the city navigation scenario from Experiment 1. Participants collected five red balls while navigating through the city using a real-time map displayed on a virtual tablet. (b) An overview of one of the cities from a bird’s eye perspective.

In the Unity game engine, we designed five different virtual cities. On arbitrary locations along the streets of each city, we placed five red balls to be collected as targets (Fig 3). The streets were 20 units wide on average, and the map of each city was exactly 254 × 254 units. We provide the target locations in https://osf.io/ne8w7. To avoid learning effects, each trial involved a different virtual city and a different placement of the red balls. While maneuvering a real vehicle on the ground or in the air (e.g., a bicycle or a hang-glider), we would typically direct our visual attention to the environment and not to the vehicle or ourselves. To avoid potential confounding effects from tracking the physical movements of the limbs, in both scenarios, no part of the virtual body was made visible to participants (see Figs 3a and 8a). Additional information regarding navigation and progress was provided to participants in real-time through a virtual tablet that was located at a fixed position right below the frontal field of view (Fig 3). To see the tablet, participants had to tilt their heads slightly downwards. The navigation information included the display of participants’ positions in the city and the locations of the red balls on a real-time map. Progress information consisted of remaining time, the number of balls collected in each stage of the experiment, and the current stage.

Several standardized questionnaires were employed for assessing various aspects of user experience. Specifically, participants completed a basic demographic questionnaire, an Immersive Tendency Questionnaire (ITQ) [48], a Presence Questionnaire (PQ) [48], a System Usability Questionnaire (SUS) [49], a NASA TLX questionnaire [50], and a Simulator Sickness Questionnaire (SSQ) [40]. The questions in these standard questionnaires were used verbatim (i.e., as documented in the corresponding references). The questionnaires were presented to the participants in separate Google Forms [51] (i.e., on a browser).

### Procedure

**Fig 4:**
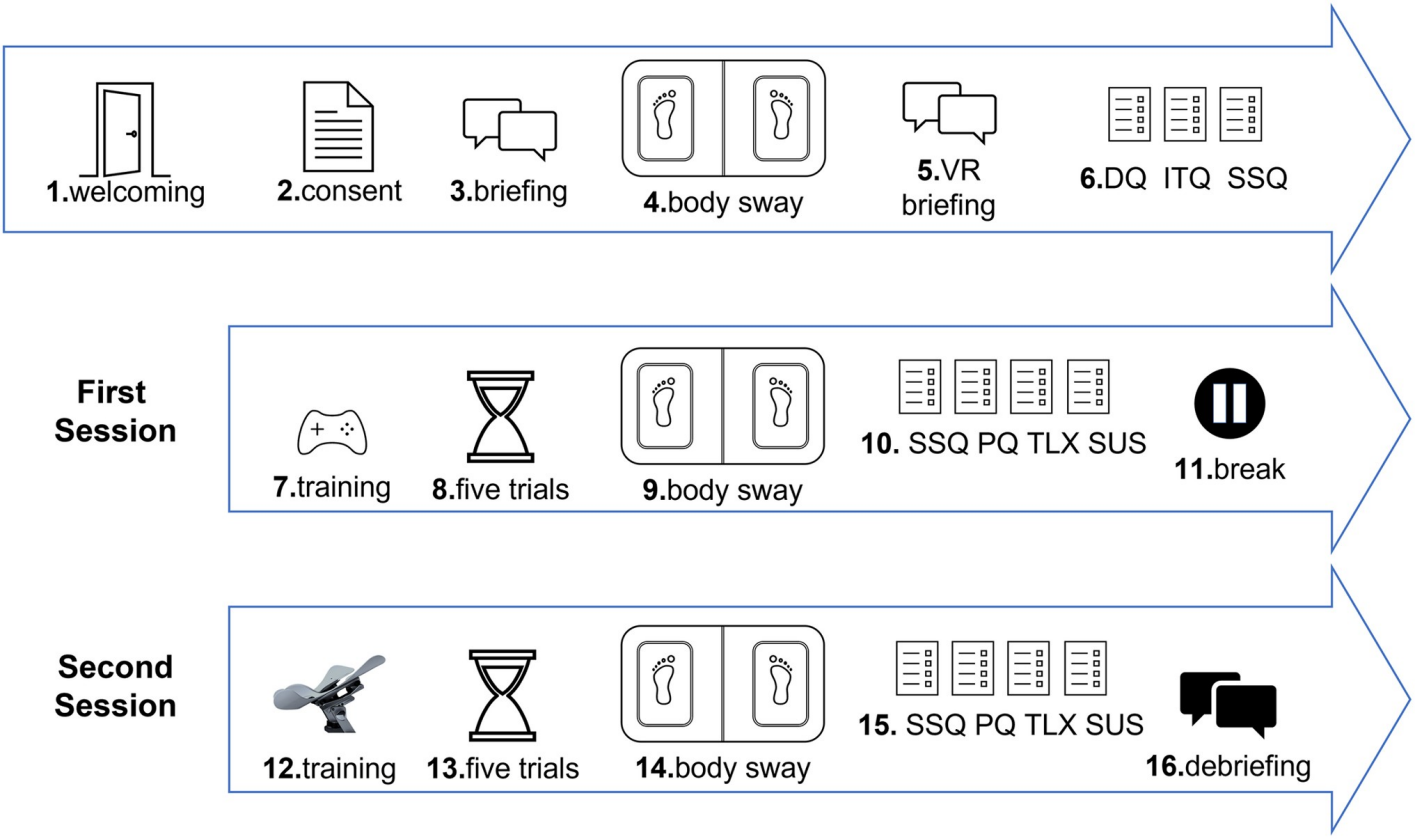
Sixteen steps of the procedure for Experiments 1 and 2. The first six steps included informed consent, baseline measurements for body sway and the questionnaires, and a general introduction to VR. The next five steps included training and testing for one of the control interfaces, and the final five steps included training and testing for the other control interface. The order of control interfaces was counterbalanced across participants.

In order to encourage focused engagement and to mitigate fatigue, participants were tested over two consecutive sessions of approximately 30 minutes each. The whole experimental procedure includes sixteen steps (Fig 4): After providing written consent, each participant was briefed on the procedure. The first session began with body sway measurements on the WBB. Participants were then briefed about the VR system (i.e., Oculus Rift, gamepad, and Limbic Chair). Then, participants completed a demographic questionnaire (DQ), ITQ, and SSQ. Next, each participant was trained to navigate using one of the control interfaces (order counterbalanced across participants). During training, each participant practiced maneuvering with the control interface and searching and collecting the balls using the interactive map. Training continued until the participant reported being comfortable with the control interface, which lasted approximately 3 minutes. After training, participants conducted five test trials with the first control interface. The maximum duration of each trial was fixed to two minutes. At the end of the first session, body sway was measured again, and the participant completed SSQ, PQ, NASA TLX, and SUS questionnaires. After a short break, participants completed another training procedure and five test trials with the second control interface. This second session ended with another body sway measurement and the SSQ, PQ, NASA TLX, and SUS questionnaires. To conclude, we asked the participants to comment about their overall experience with both control interfaces. The experimenter noted participants’ feedback and debriefed them.

### Design and analyses

The two independent variables for this experiment were the control interface (i.e., chair versus gamepad; within-subjects) and the order of conditions (i.e., participants who started with the chair versus those who started with the gamepad; between-subjects). The dependent variables for this study can be placed into three categories: questionnaires, performance measures, and additional measures.

We compared ITQ ratings between the two participant groups using a two-tailed, independent-samples t-test. Two (control interface) by two (order of conditions) mixed-design ANOVAs were used to analyze participant responses to the PQ, SUS, and NASA TLX questionnaires. For the SSQ, we subtracted baseline responses (i.e., pre-experiment) from responses obtained after using each control interface and then conducted another two (control interface) by two (order of conditions) mixed-design ANOVA. We also averaged each performance measure (i.e., time and accuracy) over five trials before running separate two (control interface) by two (order) mixed-design ANOVAs. Additional measures included body sway and head movement. Body sway was calculated as the mean distance of each measured center of mass from the mean center of mass over the course of each trial. Larger values for body sway indicated more simulator sickness. Head movements were calculated as the sum of absolute angles between subsequent head direction measurements. These additional measures were analyzed using separate two (control interface) by two (order of conditions) mixed-design ANOVAs.

**Fig 5:**
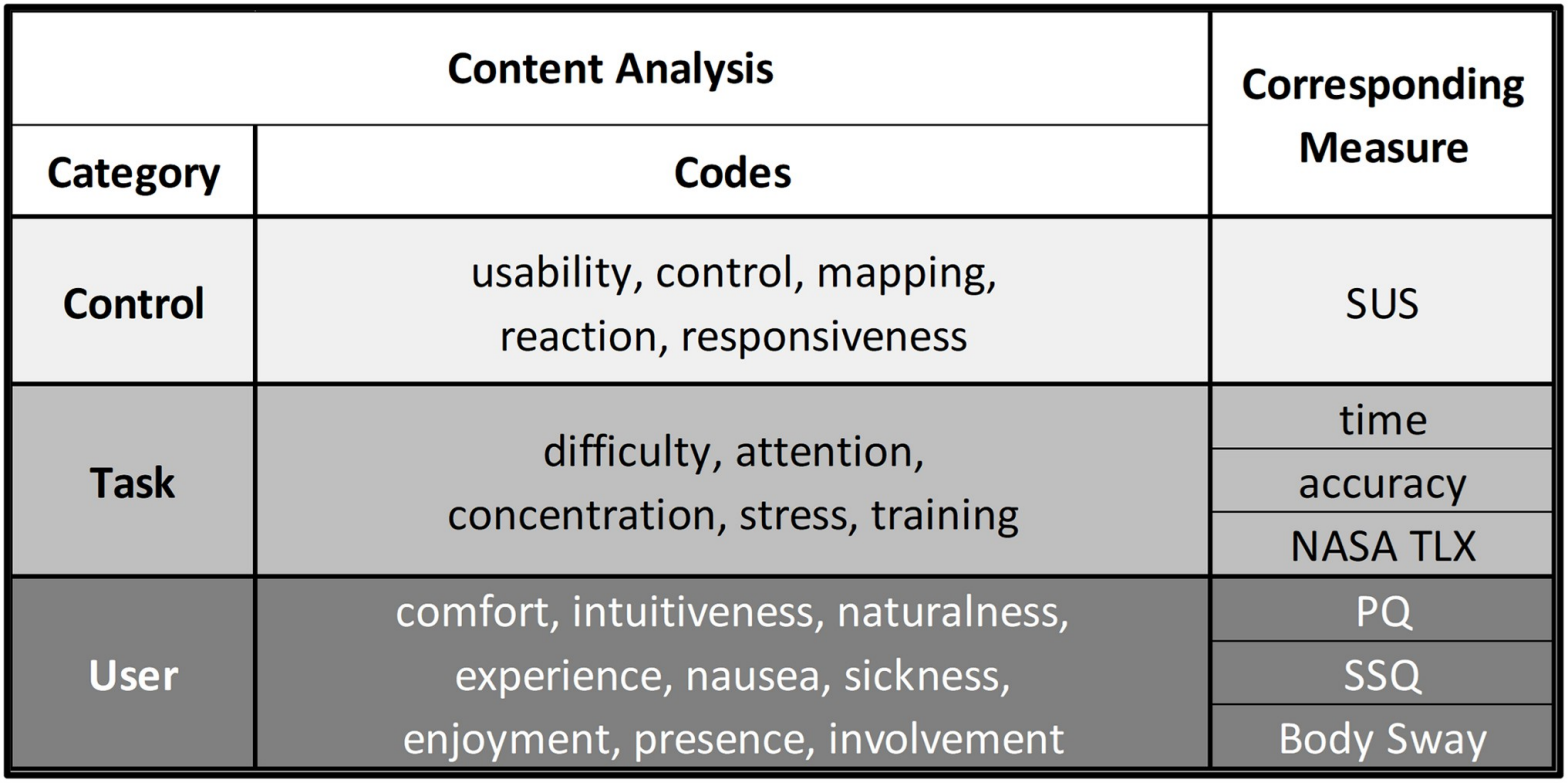
Categories and codes of the content analysis and the measures and questionnaires to which they correspond.

We also conducted content analysis to assess the oral feedback provided by participants at the end of the experiment (i.e., exit interviews). First, we transcribed all statements and sorted them into two groups (i.e., Limbic Chair and Gamepad). A sentence-by-sentence analysis allowed us to link each statement with a key concept. We identified a list of key concepts (i.e., codes) that can be grouped into three categories. The first category **Control** relates to the functional definition of each control interface and included statements referring to the effects of the control interface on the virtual environment. The codes that we linked with Control are usability, control, mapping, reaction, and responsiveness. Thus, participants’ feedback on Control can be linked with the results of the SUS. The second category **Task** relates to the effects of the control interface on the experimental task. We identified difficulty, attention, concentration, stress, and training as relevant codes for the Task category. We relate this category with the performance measures and the NASA TLX. The last category **User** is related to the effect of the control interface on user experience. The codes that we associated with User are comfort, intuitiveness, naturalness, experience, nausea, sickness, enjoyment, presence, and involvement. Therefore, we link this category with the PQ, SSQ, and body sway measurements. For each category and control interface, we counted the total number of positive and negative statements. For example, “Controller A was difficult to use” was counted as a negative statement in the User category. “With Controller B, it was easier to focus on the task” was counted as a positive statement in the Task category. Fig 5 shows how the categories and codes of the content analysis relate to the user experience and performance measurements.

### Results

**Fig 6:**
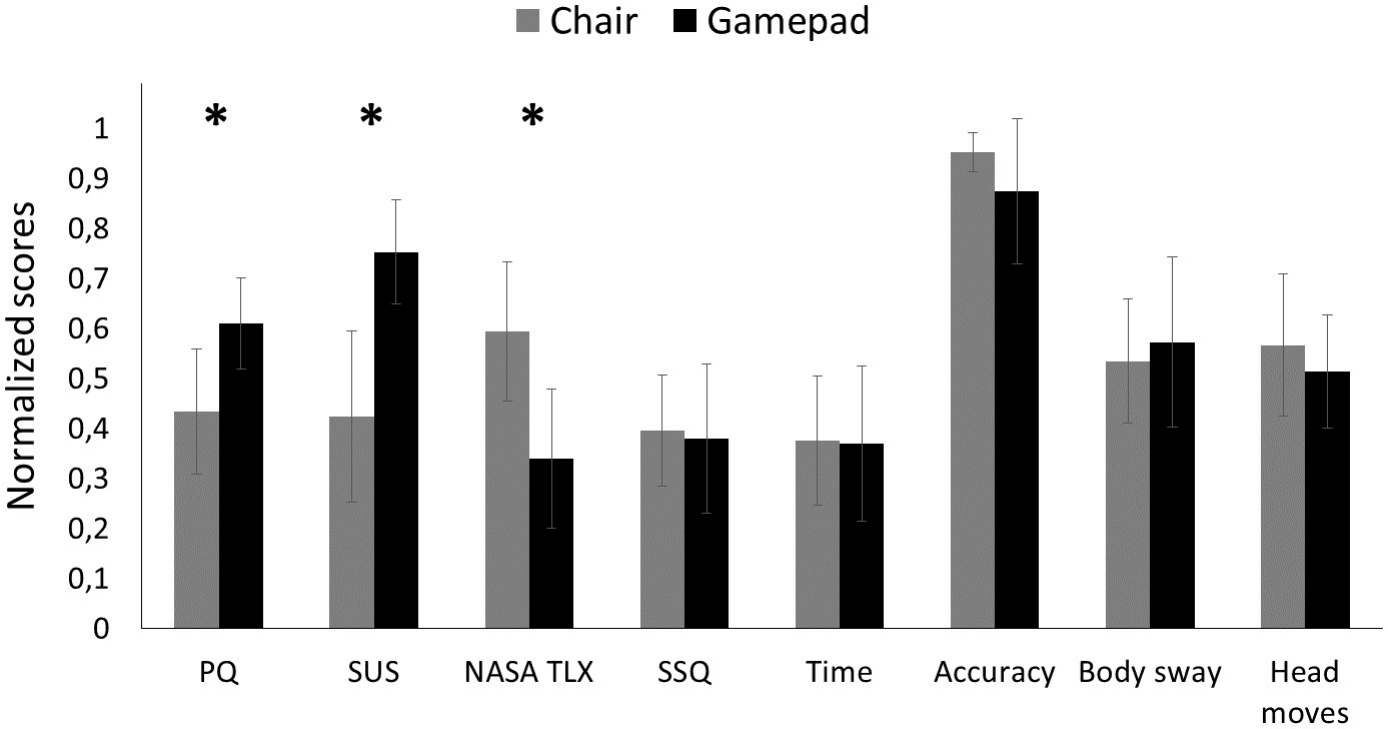
Bar chart of all dependent measures from Experiment 1. For this visualization only, each dependent measure was normalized to a value between 0 and 1 by subtracting the minimum value from each individual value and then dividing by the maximum value. Error bars represent ±2 standard errors of the mean (SEMs). Asterisks mark a significant main effect of control interface. Larger values are desirable for the presence questionnaire (PQ), system usability scale (SUS), and Accuracy. Smaller values are desirable for workload (NASA TLX), simulator sickness questionnaire (SSQ), Time, and Body sway.

In Fig 6, we present the comparison between control interfaces for each dependent measure in Experiment 1. We deliberately present normalized scores to simplify comparisons among scores and across experiments. For the text below, we also provide descriptive statistics for the original scores to facilitate comparison with the general literature.

There was no statistically significant difference between participant groups in terms of ITQ, *t* = 0.46, *se* = 8.43, *p* = 0.66, indicating that participants who started with the Limbic Chair had comparable tendencies to be immersed in virtual environments as those who started with the gamepad. The inferential statistics for the questionnaire results from Experiment 1 are listed in Table 1. The ANOVA for PQ did not reveal a main effect of order or an interaction. However, self-reported presence was higher for the gamepad (*M* = 118.3, *SD* = 15.2) than the chair (*M* = 102.6, *SD* = 20.9), resulting in a main effect of control interface. The ANOVA for SUS did not reveal a main effect of order, but there was a significant main effect of control interface and a significant interaction, revealing that the gamepad (*M* = 83.9, *SD* = 12.7) was rated higher than the chair (*M* = 62.5, *SD* = 20.8), especially when the gamepad was tested before the chair. The ANOVA for NASA TLX did not reveal a main effect of order or an interaction, but self-reported workload was lower for the gamepad (*M* = 4.5, *SD* = 1.23) than the chair (*M* = 5.7, *SD* = 1.23), resulting in a main effect of control interface. The ANOVA for the SSQ did not reveal a main effect of control interface, a main effect of order, or an interaction.

**Table 1:** Questionnaire results from Experiment 1. Significant results (*p* < 0.05) are bold.

2 × 2 ANOVA	PQ	SUS	NASA TLX	SSQ
Order	*F*(1,12) = 0.34	*F*(1,12) = 2.30	*F*(1,12) = 1.01	*F*(1,12) = 0.16
	MSE = 552.98	MSE = 262.05	MSE = 2.54	MSE = 1040.08
	*p* = 0.57	*p* = 0.16	*p* = 0.34	*p* = 0.70
Controller	*F*(1,12) = 12.12	*F*(1,12) = 15.16	*F*(1,12) = 19.63	*F*(1,12) = 0.11
	MSE = 142.57	MSE = 212.05	MSE = 0.52	MSE = 169.18
	***p* < 0.01**	***p* < 0.01**	***p* < 0.01**	*p* = 0.75
Controller × Order	*F*(1,12) = 0.90	*F*(1,12) = 6.74	*F*(1,12) = 0.51	*F*(1,12) = 0.96
	MSE = 142.57	MSE = 212.05	MSE = 0.52	MSE = 169.18
	*p* = 0.36	***p* = 0.02**	*p* = 0.49	*p* = 0.35

In Table 2, we report the inferential statistics for performance scores (i.e., time and accuracy), body sway, and head movements. The ANOVA for performance scores did not reveal a main effect of control interface or a main effect of order. However, there was a significant interaction that suggested that the control interface that was used second led to better performance (i.e., participants improved over time). The ANOVA for body sway did not reveal a main effect of control interface, a main effect of order, or an interaction. The ANOVA for head movements did not reveal a main effect of control interface, a main effect of order, or an interaction.

**Table 2:** Analysis of performance and additional measures from Experiment 1. Significant results (*p* < 0.05) are bold.

2 × 2 ANOVA	Time	Accuracy	Body Sway	Head Movements
Order	*F*(1,12) = 1.2	*F*(1,12) = 2.36	*F*(1,12) = 0.77	*F*(1,12) = 0.24
	MSE = 157.14	MSE = 0.15	MSE = 0.10	MSE < 0.01
	*p* = 0.3	*p* = 0.15	*p* = 0.40	*p* = 0.64
Controller	*F*(1,12) = 0.02	*F*(1,12) = 2.34	*F*(1,12) = 0.35	*F*(1,12) = 0.39
	MSE = 14.0	MSE = 0.06	MSE = 0.02	MSE < 0.01
	*p* = 0.9	*p* = 0.15	*p* = 0.56	*p* = 0.55
Controller × Order	*F*(1,12) = 44.6	*F*(1,12) = 6.0	*F*(1,12) = 0.26	*F*(1,12) = 0.01
	MSE = 14.0	MSE = 0.06	MSE = 0.02	MSE < 0.01
	***p* < 0.01**	***p* = 0.03**	*p* = 0.62	*p* = 0.94

**Fig 7:**
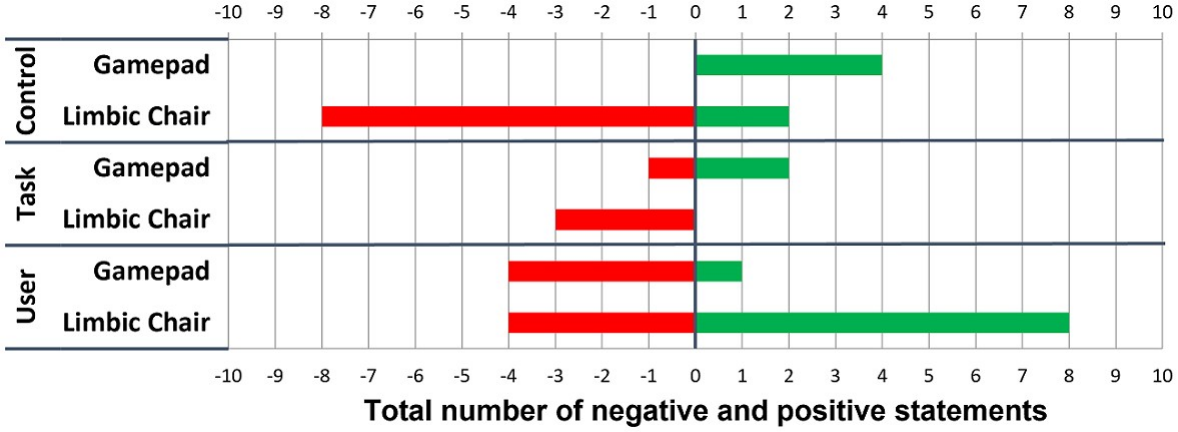
The results of the content analysis from Experiment 1. The total numbers of negative (red) and positive (green) statements are shown on the horizontal axis and encoded in three categories (Control, Task, and User) and for each control interface (vertical axis).

For both control interfaces, Fig 7 shows a histogram of the total number of negative and positive statements that are encoded into Control, Task, and User categories. Previous work [22, 32] has suggested that providing segments of actual data such as participants’ quotes improves the interpretation of such qualitative data [52]. Therefore, we explain the results of the content analysis in combination with representative quotes from our participants, where [Px] denotes the participant number. Participants reported that the gamepad allowed for more **Control** of movement through the virtual environment than the Limbic Chair because “the moves are more intuitive” [P5] and “the chair was difficult to figure out” [P18]. The gamepad seems to have a more positive effect on solving the **Task** because “it takes time to get used to the chair” [P4]. Participants reported their **User** experience with the gamepad rather negatively: “With the gamepad you actually do not move but you move virtually.” [P9]. On the other hand, there were twice as many positive statements than negative statements about the Limbic Chair for the User category. Many participants found the Limbic Chair to be “more natural” [P9], “more immersive” [P6], and “more real life-like” [P3], while only few found it “not natural” [P15] and “not intuitive” [P2].

In summary, the gamepad seems to be the more suitable control interface for the city navigation task. This pattern may be due to familiarity, represented by the quote “I am more used to joysticks than to using my legs” [P5]. On the other hand, users’ VR experience was reported as “much more engaging with the chair” [P3]. “I had one moment with the chair where I felt immersed while I was moving forward and turning at the same time. It was cool. It did not happen with the gamepad” [P6]. However, “the chair requires more effort” [P2, P5]. Thus, the comments on the Limbic Chair suggest that improved user experience comes with the cost of extra physical effort.

## Experiment 2: Flight simulation

In Experiment 2, we investigated whether we could replicate the results of Experiment 1 for another common VR scenario. Inspired by Evans and Sutherland’s demonstration of *Los Angeles 2000* at ACM SIGGRAPH ‘93 [53, 54], we employed flight simulation with a virtual hang-glider for Experiment 2. A live demonstration of the Limbic Chair system with the flight simulation scenario was presented as part of ACM SIGGRAPH 2018 [55]. In this experiment, participants were asked to fly through rings that are placed in the air (i.e., primary task). While doing so, participants were also requested to count the total number of the birds (i.e., secondary task) that they see flying in the environment. The motivation behind this dual-task setup was to test whether differences between the embodied and non-embodied control interfaces in terms of posture would affect freedom of head movement. Similar to Experiment 1, the overall goal of Experiment 2 was to compare the effects of two control interfaces on performance and user experience.

### Participants

Twenty-two people participated in Experiment 2 and received 25 CHF compensation. The data from 20 participants (9 female and 11 male, aged 22 to 43) were included in the final analyses because two datasets were incomplete. The participants had normal or corrected-to-normal vision and were informed about the potential risks of simulator sickness. The participants had no prior experience with the Limbic Chair, but all of them had some experience with a gamepad.

### Apparatus and materials

In Experiment 2, we used the same VR system as in Experiment 1. However, the mappings of pitch and yaw movements of both the joystick and Limbic Chair were adjusted to the flight simulation scenario. The mappings from joystick and Limbic Chair movements to side-to-side (yaw) and up-and-down (pitch) movements of the virtual hang-glider are detailed in Eqs 3 and 4:

$$\begin{array}{c} \begin{array}{c} pitch=25\times Joy_{y}\\ yaw_{i+1}=yaw_{i}+50\times Joy_{x}\times dT \end{array} \end{array}$$ (3)

$$\begin{array}{c} \begin{array}{c} pitch=4\times (LeftLeg_{y}+RightLeg_{y})\\ yaw_{i+1}=yaw_{i}+4\times \left(LeftLeg_{x}+RightLeg_{x}\right)\times dT \end{array} \end{array}$$ (4)

The subscripts *y* and *x* in Eqs 3 and 4 correspond to pitch and yaw rotations, respectively, of the gamepad and the Limbic Chair in degrees. It is important to note that, during city navigation, the pitch movements of both controllers were mapped to the forward movements of the vehicle. In the flight simulation, the forward speed of the hang-glider was kept constant at 80 units per second, whereas the pitch movement of both controllers were directly mapped to the pitch movement of the hang-glider. Also here, the constant multipliers in Eqs 3 and 4 (respectively 25, 50 and 4) were determined empirically based on pilot testing.

**Fig 8:**
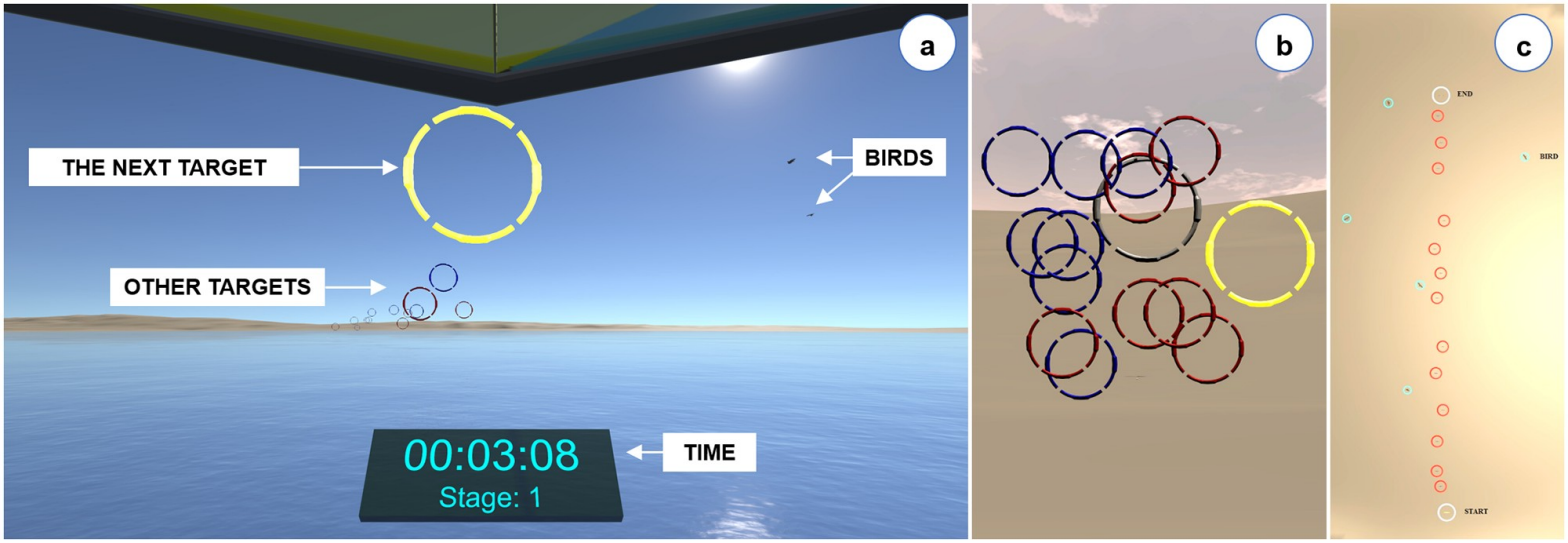
(a) A screenshot of the flight simulation scenario from Experiment 2. Participants flew through fifteen rings and counted the number of birds. The next target ring was highlighted in yellow. A series of rings shown from (b) orthographic-frontal and (c) top views.

For Experiment 2, we generated five different flight routes. In the Unity game engine, we defined each route by 15 rings that were arbitrarily positioned in the air at variable intervals (Fig 8). The flight distance for each route was 7500 (±233.33) units. Thus, the total flight time would take 7500 (±233.33) units / (80 units/s) = 93.75 (±2.92) seconds, assuming the user flew in a straight line to the finish (i.e., the last ring). With respect to this straight line, the rings were distributed within a range of ±300 horizontal and 30 to 200 vertical units. The virtual environment also included randomly generated flying birds on the right and left of the flight routes. To avoid potential learning effects, the constellation of rings and the total number of birds varied from trial to trial.

### Procedure

In Experiment 2, participants followed the same procedure (including a training session for approximately 3 minutes) as in Experiment 1 (Fig 4), except that they were asked to count the number of birds while flying through the virtual environment. For each missed ring, a 10-second penalty was added to the time score. The time for each trial began automatically when the participant traversed the first ring and then stopped when the participant traversed the last ring.

### Design and analyses

The design of Experiment 2 was similar to Experiment 1. Here, *time* refers to the mean time required to complete a trial, and *accuracy* refers to the number of rings successfully traversed. Experiment 2 also included the additional dependent measure of bird count (i.e., the number of birds that the participant reported seeing). Time, accuracy, and bird count were all analyzed using separate two (control interface) by two (order) mixed design ANOVAs. In Experiment 2, we assessed the oral feedback provided by the participants with the same content analysis procedure from Experiment 1.

### Results

**Fig 9:**
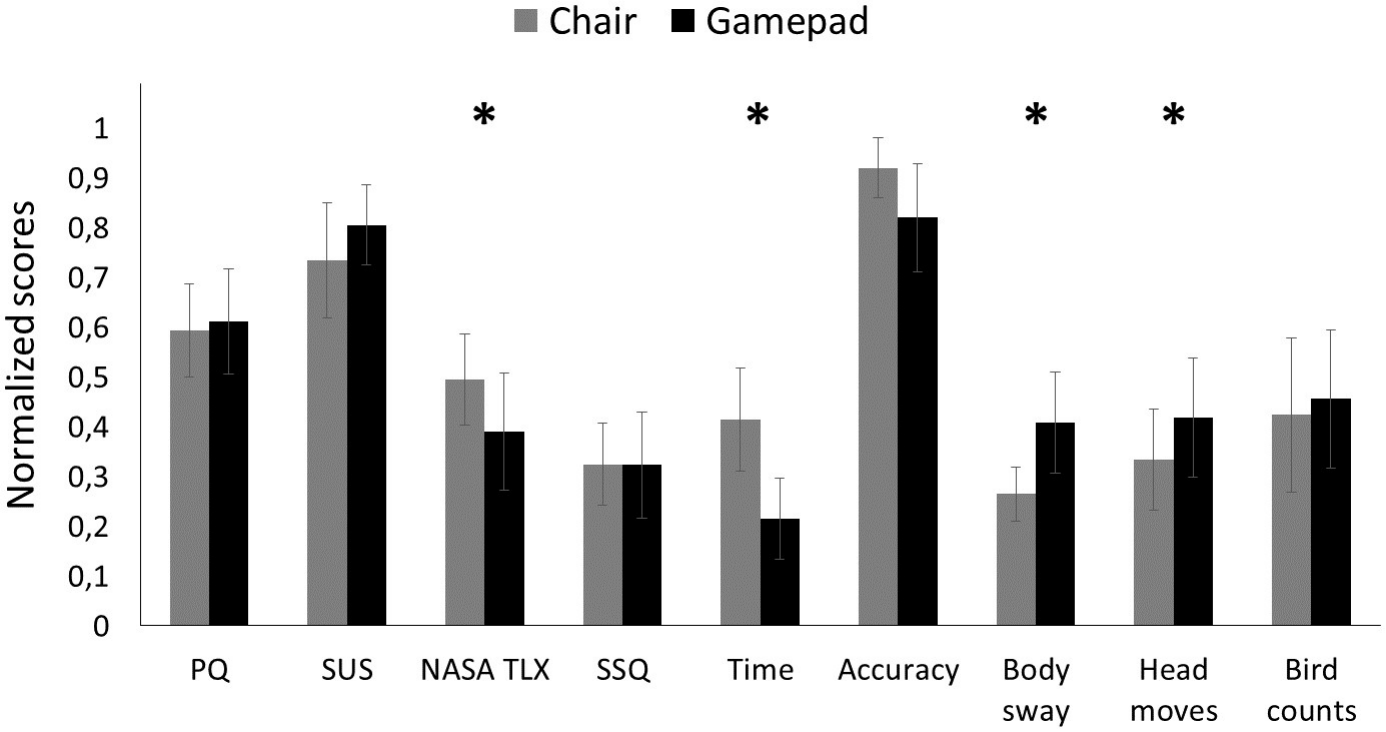
Bar chart of all dependent measures from Experiment 2. For this visualization only, each dependent measure was normalized to a value between 0 and 1 by subtracting the minimum value from each individual value and then dividing by the maximum value. Error bars represent ±2 SEMs. Asterisks denote a significant main effect of control interface. Larger values are desirable for the presence questionnaire (PQ), system usability scale (SUS), Accuracy, and Bird counts. Smaller values are desirable for workload (NASA TLX), simulator sickness questionnaire (SSQ), Time, and Body sway.

Comparisons between the control interfaces for each dependent measure from Experiment 2 are illustrated in Fig 9. There was no statistically significant difference between the different participant groups in terms of ITQ, *t* = 0.80, *se* = 5.52, *p* = 0.44, suggesting that participants in both groups had comparable tendencies to be immersed in virtual environments. The inferential statistics for the questionnaire results from Experiment 2 are listed in Table 3. The ANOVA for PQ did not reveal main effects for either control interface or order, nor an interaction. The ANOVA for SUS did not reveal a main effect of either control interface or order. However, there was a significant interaction for SUS, suggesting that the control interface used first was rated as more usable than the control interface used second. The ANOVA for NASA TLX did not reveal a main effect of order, but there was a significant main effect of control interface and a significant interaction. The NASA TLX results demonstrate that participants rated workload to be higher for the chair (*M* = 6.06, *SD* = 1.5) than the gamepad (*M* = 5.32, *SD* = 1.9), especially when the gamepad was tested before the chair. The ANOVA for SSQ did not reveal a main effect of either control interface or order. However, there was a significant interaction in which the control interface used second led to more self-reported simulator sickness than the control interface used first.

**Table 3:** Questionnaire results from Experiment 2. Significant results (*p* < 0.05) are bold.

2 × 2 ANOVA	PQ	SUS	TLX	SSQ
Order	*F*(1,18) = 0.855	*F*(1,18) = 1.69	*F*(1,18) < 0.01	*F*(1,18) = 0.06
	MSE = 286.46	MSE = 379.51	MSE = 5.18	MSE = 2660.64
	*p* = 0.37	*p* = 0.21	*p* > 0.99	*p* = 0.812
Controller	*F*(1,18) = 0.071	*F*(1,18) = 1.46	*F*(1,18) = 9.37	*F*(1,18) < 0.01
	MSE = 257.79	MSE = 289.51	MSE = 0.59	MSE = 312.58
	*p* = 0.8	*p* = 0.24	***p* = 0.01**	*p* = 0.97
Controller × Order	*F*(1,18) = 3.538	*F*(1,18) = 8.84	*F*(1,18) = 4.76	*F*(1,18) = 14.29
	MSE = 257.79	MSE = 289.51	MSE = 0.59	MSE = 312.58
	*p* = 0.08	***p* = 0.01**	***p* = 0.04**	***p* < 0.01**

Table 4 summarizes the time, accuracy, body sway, head movement, and bird count results. The ANOVA for time did not reveal a main effect of order or an interaction. However, the gamepad led to significantly faster completion times (*M* = 84.05 seconds, *SD* = 0.72) than the chair (*M* = 84.84 seconds, *SD* = 0.92), resulting in a main effect of control interface. The ANOVA for accuracy did not reveal a main effect of either control interface or order, nor an interaction. The ANOVA for body sway did not reveal a main effect of order or an interaction. However, participants exhibited less body sway after using the chair (*M* = 0.06, *SD* = 0.09) than after using the gamepad (*M* = 0.17, *SD* = 0.17), resulting in a main effect of control interface. The ANOVA for head movements did not reveal a main effect of order or an interaction. However, the gamepad led to more head movements (*M* = 0.44, *SD* = 0.17) than the chair (*M* = 0.38, *SD* = 0.15), resulting in a main effect of control interface. The ANOVA for bird counts did not reveal a main effect of either control interface or order, nor an interaction.

**Table 4:** Analysis of performance and additional measures from Experiment 2. Significant results (*p* < 0.05) are bold.

2 × 2 ANOVA	Time	Accuracy	Body Sway	Head Movements	Bird Count
Order	*F*(1,18) = 0.09	*F*(1,18) = 0.30	*F*(1,18) = 0.40	*F*(1,18) = 0.96	*F*(1,18) = 0.43
	MSE = 1.31	MSE < 0.01	MSE = 0.03	MSE = 0.05	MSE = 2.22
	*p* = 0.77	*p* = 0.59	*p* = 0.53	*p* = 0.34	*p* = 0.52
Controller	*F*(1,18) = 48.03	*F*(1,18) = 3.72	*F*(1,18) = 8.98	*F*(1,18) = 5.09	*F*(1,18) = 0.36
	MSE = 0.13	MSE < 0.01	MSE = 0.01	MSE = 0.01	MSE = 0.34
	***p* < 0.01**	*p* = 0.07	***p* = 0.01**	***p* = 0.04**	*p* = 0.56
Controller × Order	*F*(1,18) = 0.22	*F*(1,18) = 0.6	*F*(1,18) = 2.38	*F*(1,18) = 0.48	*F*(1,18) = 0.24
	MSE = 0.13	MSE < 0.01	MSE = 0.01	MSE = 0.01	MSE = 0.34
	*p* = 0.64	*p* = 0.45	*p* = 0.14	*p* = 0.50	*p* = 0.63

**Fig 10:**
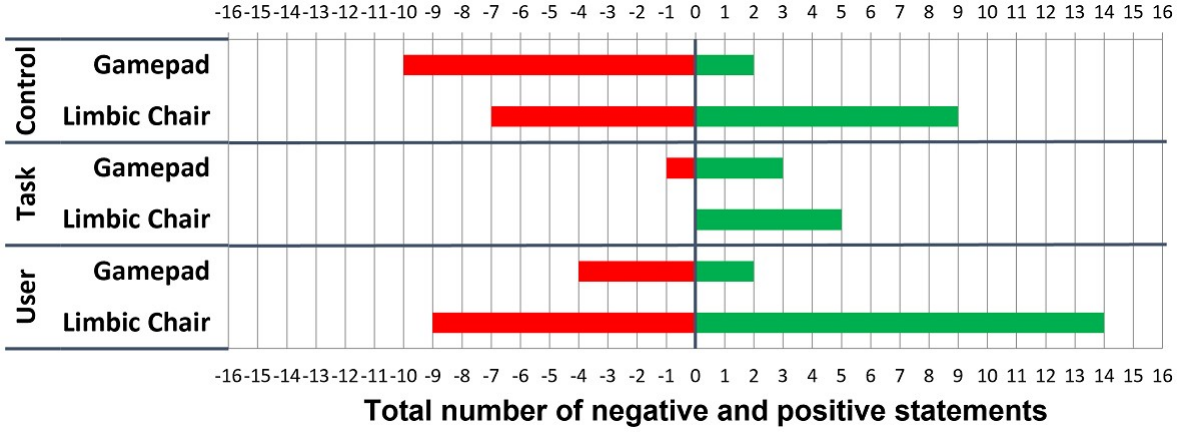
The content analysis of Experiment 2. The total numbers of negative (red) and positive (green) statements are shown on the horizontal axis and encoded in three categories (Control, Task, and User) and for each control interface (vertical axis).

Fig 10 depicts the content analysis of the exit interviews. Participants reported that the gamepad did not provide optimal **Control** of movement through the virtual environment because movements using the gamepad were “changing fast” [P2, P5, P7, P8], “jerky” [P9], snapping back to the center and causing “fast jumps” [P11], or “discrete” [P22]. On the other hand, participants had a split opinion on the Limbic Chair’s usability as a control interface. Some participants found that the control and movements with the chair were much “smoother” [P7, P9], while others found that the chair’s “stiffness and height should be adjusted for the user” [P14]. For solving the **Task**, the Limbic Chair was associated with more positive comments than the gamepad. Overall, some participants found the chair “easier” for the task [P2, P4, P5, P13], while others favored the gamepad [P3, P21]. The comments for both control interfaces in terms of **User** experience were approximately evenly distributed. For some participants, the Limbic Chair was more “natural” [P17] and “enjoyable” [P9, P13] to use, while others found the gamepad “natural” to use [P6, P16]. Overall, participants provided more comments about the Limbic Chair than the gamepad.

In summary, for the flight simulation scenario, the Limbic Chair seems to be the more suitable control interface than the gamepad: “Body control gives the feeling of VR” [P19], “I am actually sensitive to simulator sickness and my experience was very comfortable with the chair” [P7], “You feel like you are more in the game with the chair with respect to the gamepad” [P20].

## Discussion

In this paper, we pursued two goals. First, we introduced a novel embodied interface called the Limbic Chair. Then, in two experimental scenarios, we studied the following research question: How suitable is the Limbic Chair for tasks in virtual environments that are typically performed with conventional control interfaces? Concretely, we compare the Limbic Chair to a gamepad using five measures: presence, system usability, work load, simulator sickness, and performance (i.e., time and accuracy). In the city navigation scenario, the gamepad was associated with better presence, usability, and workload scores. In the flight simulation scenario, the chair was associated with less simulator sickness but slower performance and higher workload. Below, we discuss the potential implications of our findings for each of these measures and the limitations of our study.

### Presence

According to Witmer and Singer, presence is defined as the subjective experience of being in a virtual environment despite being physically located in a different environment. Presence in a virtual environment can be affected by mediation between the user and the environment by the control interface [48]. More “natural” control interfaces could affect how users inhabit a virtual environment and should lead to more presence (i.e., inhabito, ergo sum).

In our city navigation experiment, the gamepad led to higher presence scores than the Limbic Chair. This finding is consistent with Kitson and colleagues [22] but contradict other studies that have found that embodied interfaces have led to more presence [5, 9]. Although the city navigation experiment from Kitson and colleagues [22] was similar to ours, their design included only one questionnaire in which participants rated their agreement with individual statements related to presence and other factors. Kitson and colleagues [22] acknowledge that this finding may have resulted from a lack of communication with the participants regarding the definition of presence. Despite using the standard Presence Questionnaire [48] in the present study, our findings are consistent with Kitson and colleagues [22], suggesting that our specific city navigation task may explain the differences between studies on embodied interfaces in terms of presence.

Our findings suggest that the experimental design as well as the type of task have an impact on to what extent an embodied control interface can induce presence in a virtual environment. Although presence was significantly lower for the Limbic Chair compared to the gamepad in the city navigation task, in the flight simulation task, the average presence score with the Limbic Chair was similar to the gamepad (approximately 0.6). The content analysis revealed that, in the flight simulation, experiences with the Limbic Chair were reported as more “natural” than experiences with the gamepad. In this second experiment, the pitch movement of the controllers were directly mapped to the pitch movement of the hang-glider in 3D space. This difference in mappings between the two experiments was necessary because of the spatial dimensionality of the tasks. However, these findings indicate that the use of embodied interfaces may be task-dependent (cf. [5]).

### System usability

The usability ratings of the two control interfaces also differed between the two experimental scenarios. The usability scores for the gamepad were similar across experiments (i.e., 83.9 for city navigation and 77.5 for flight simulation), but the usability scores for the Limbic Chair were substantially lower for the city scenario (62.5) compared to the flight simulation scenario (71.0). This finding suggests that the usability of the Limbic Chair was affected by the underlying experimental task, but future research should systematically vary experimental task for the same participants in order to verify this pattern. The order of control interfaces also affected usability ratings. In the city navigation scenario, the gamepad was rated as more usable when participants used the gamepad before the Limbic Chair. This finding further supports the claims that familiarity with a control interface influences usability (consistent with [5, 9, 22]) and that embodied interfaces may be found less usable than non-embodied interfaces if they are also less familiar [9, 22, 28].

### Work load

In both experimental scenarios, the gamepad led to lower overall workload. This finding might be attributable to the facts that the Limbic Chair required more body movement and was less familiar than the gamepad. Indeed, during the exit interviews, some participants reported that the Limbic Chair provided an improved VR experience but required more effort than the gamepad. Similar findings were reported in other user experiments [5, 9, 22, 28] in which the participants who preferred a conventional handheld interface over an embodied interface found that the latter requires more concentration, physical effort, and familiarity. According to Jacob and colleagues [26], the realism provided by an interface (i.e., presence) can be sacrificed in order to reduce mental or physical effort, especially when the interface is unfamiliar to the user. Future research on control interfaces should focus on this possibility by systematically testing the effect of familiarity on the relationship between realism and workload.

### Simulator sickness

According to previous research [38, 39], postural instability measured by body sway is a reliable predictor of simulator sickness. In both of the present experiments, we measured our participants’ postural stability with the WBB immediately after they were exposed to the virtual environment. Following the body sway measurement, participants were asked to complete the SSQ. In the city navigation scenario, the two control interfaces had similar effects on simulator sickness in terms of both SSQ and body sway. However, in the flight simulation scenario, the gamepad led to significantly more body sway than the Limbic Chair, although the SSQ results for the two control interfaces were not significantly different. Our SSQ measurements may have been less sensitive to the difference between control interfaces than the body sway measurements because the body sway measurements were recorded immediately after exposure to VR and before participants completed the SSQ.

In recent years, there is a critical discussion around the use of SSQ as an established and appropriate measure of discomfort in virtual environments. Several alternative simulator sickness questionnaires have been proposed (e.g., VRSQ [56] and CSQ [57]), although previous research has suggested that these two newer questionnaires are similar to the SSQ in terms of sensitivity [58]. In a recent work, Hirzle and colleagues [59] systematically reviewed the use of SSQ in VR research over the last decade. Their results show that the SSQ addresses only some of the factors that affect overall discomfort in VR, although it is extensively used. Hirzle and colleagues call for an update of the SSQ or a new, more comprehensive, questionnaire that would include additional factors such as eye strain and ergonomics.

In addition, Hodgson and colleagues reported that simulator sickness can be higher in virtual environments with additional environmental structures compared to an open field [60]. This principle could also explain the difference we observed between the city navigation and flight simulation tasks in terms of simulator sickness because the virtual city contained many environmental structures (Fig 3) whereas the flight simulation occurred in an open sky (Fig 8).

Compared to the calibration sample of SSQ scores provided by [40], SSQ scores for both of the present experiments were relatively high. SSQ scores were in the 90th percentile for the city navigation scenario and in the 85th percentile for the flight simulation scenario. In addition, independent of which control interface was used first, participants’ SSQ scores increased over the course of the experiment. Taken together, these findings suggest that some symptoms of simulator sickness (i.e., body sway) were not detected with the SSQ in the flight simulation scenario. Indeed, our body sway results support the hypothesis that the Limbic Chair causes less simulator sickness than the gamepad because the gamepad causes more postural instability than the Limbic Chair.

### Performance

For city navigation, there was no significant effect of the control interface on task completion time. However, over the course of the experiment, completion times decreased. For flight simulation, the gamepad led to faster performance than the Limbic Chair. In both experiments, performance accuracy for the two control interfaces was similar.

In previous studies, researchers reported that users’ familiarity with handheld interfaces led to better performance than embodied interfaces [5, 9]. When using a familiar interface such as a gamepad or joystick, users can dedicate most of their attention to the task at hand. Conversely, when the interface is unfamiliar, users need to split their attention between using the interface and solving the task. In the city navigation scenario, participants’ lower familiarity with the Limbic Chair did not affect their performance, but in the flight simulation scenario, participants’ familiarity with the gamepad may have helped them focus on piloting the hang-glider.

Another possible explanation for performance differences in the flight simulation scenario is the amount of head movement that was required for participants to complete the task. In the flight simulation scenario, participants had to turn their heads to the sides to see and count the virtual birds while piloting the hang-glider. Participants moved their heads significantly more while using the gamepad than while using the chair, but their counting accuracy remained similar with the two control interfaces. Unfortunately, the present results do not allow us to distinguish between the possibilities that head movements indicated the availability of attentional resources or more distraction as a result of using the gamepad compared to the chair. Future research should systematically test whether there can be a relationship between head movements and performance during a visual search task in 3D virtual environments.

While embodied interfaces may be intuitively appealing, they should be selected for specific types of tasks and users. Even when an embodied interface provides more natural interaction with the virtual environment, users might need adequate training with the more novel control interface. Longitudinal studies with the Limbic Chair would be necessary to assess whether movement through a virtual environment can be improved in terms of common performance metrics (e.g., task completion time, accuracy).

### Limitations and future work

One limitation of the present user evaluation is that we only employed one particular mapping between users’ leg movements on the Limbic Chair and their corresponding movements through the virtual environment. This mapping may not be optimal for these particular tasks. As suggested by others [5, 9, 22], calibrating an embodied interface based on user anatomy and reaction time might also improve task performance and VR experience. According to our observations, the shortest and tallest participants had difficulties using the Limbic Chair. Consequently, more generic interfaces such as the gamepad may be suitable for a broader user group.

Another potential limitation of the present experiments is that participants were much more familiar with the gamepad than the Limbic Chair, which may have contributed to the differences we observed. Our evaluation may also be limited because the control interface mappings differed between city navigation (2D navigation on a planar surface) and flight simulation (3D navigation in the air). Although these mapping differences were necessitated by the spatial dimensionality of the tasks, future work could explicitly and systematically disentangle the effects of these mappings and types of tasks. In future work, researchers can also train participants to use an embodied interface such as the Limbic Chair until they reach a particular learning criterion (e.g., to reach a comparable task completion time) that matches performance with a handheld control interface. Such a training study would allow researchers to investigate the time course of training on a novel interface, as well as more directly compare an embodied and non-embodied interface while controlling for familiarity.

The extent to which embodiment can explain differences in locomotion performance between two interfaces could also be improved with a standardized questionnaire that specifically measures embodiment in terms of sense of self-location, agency, and body ownership. Recently, Gonzalez-Franco and colleagues have developed an embodiment questionnaire based on these three factors [25, 61]. While this work defines embodiment as specifically the perceived occupation of an avatar’s body, the field of embodied cognition in general emphasizes the fact that embodiment should not be considered independently of the actions with which the body is engaged to solve a task [62–64]. Our results also indicate that the benefits of embodied interfaces vary across tasks, suggesting that a standardized questionnaire on embodiment in VR should account for task differences.

## Conclusion

The present paper contributes to our knowledge of embodied interfaces in at least two ways. We show that leg movements performed during sitting are promising in their usefulness for VR applications. Our research also suggests that embodied interfaces might benefit from customization for specific tasks and users. Unfortunately, an adoption of the control interface to the structure and function of the human body does not guarantee better performance and user experience by itself because users may need time to first become familiar with the control interface and the task at hand. We therefore suspect that the Limbic Chair may provide greater benefits in future VR applications when users receive extensive training beforehand. Improvements in performance could also be achieved by tailoring the Limbic Chair to individuals’ physical characteristics and previous experience, as well as the task they need to solve in the virtual environment.

In this work, we aimed for a systematical assessment of the suitability of a new embodied control interface, the Limbic Chair, for navigation tasks that are performed in VR. Previous research has assessed similar interfaces or developed standardized questionnaires that consider a range of factors related to user experience (e.g., presence, usability) and performance (e.g., task completion time and accuracy). However, they tend not to emphasize the importance of the task at hand. Our findings suggest careful consideration of the impact of tasks for future assessments of embodied control interfaces.
